# PM_2.5_ Exposure Inhibits Transepithelial Anion Short-circuit Current by Downregulating P2Y2 Receptor/CFTR Pathway

**DOI:** 10.7150/ijms.96777

**Published:** 2024-07-22

**Authors:** Xiaolong Liu, Zhangwen Li, Jiajie Shan, Fang Wang, Zhongpeng Li, Shaohua Luo, Jian Wu

**Affiliations:** 1 Second Department of Elderly Respiratory, Guangdong Provincial People's Hospital (Guangdong Academy of Medical Sciences), Southern Medical University, 510080, Guangzhou, China.; 2 School of Medicine, South China University of Technology, Guangzhou, 510000, China.

**Keywords:** fine particulate matter, short-circuit current, P2Y2 receptor, cystic fibrosis transmembrane regulator, asthma

## Abstract

Fine particulate matter (PM_2.5_) can damage airway epithelial barriers. The anion transport system plays a crucial role in airway epithelial barriers. However, the detrimental effect and mechanism of PM_2.5_ on the anion transport system are still unclear. In this study, airway epithelial cells and ovalbumin (OVA)-induced asthmatic mice were used. In transwell model, the adenosine triphosphate (ATP)-induced transepithelial anion short-circuit current (I_sc_) and airway surface liquid (ASL) significantly decreased after PM_2.5_ exposure. In addition, PM_2.5_ exposure decreased the expression levels of P2Y2R, CFTR and cytoplasmic free-calcium, but ATP can increase the expressions of these proteins. PM_2.5_ exposure increased the levels of Th2-related cytokines of bronchoalveolar lavage fluid, lung inflammation, collagen deposition and hyperplasisa of goblet cells. Interestingly, the administration of ATP showed an inhibitory effect on lung inflammation induced by PM_2.5_. Together, our study reveals that PM_2.5_ impairs the ATP-induced transepithelial anion I_sc_ through downregulating P2Y2R/CFTR pathway, and this process may participate in aggravating airway hyperresponsiveness and airway inflammation. These findings may provide important insights on PM_2.5_-mediated airway epithelial injury.

## Introduction

Air pollution has raised a worldwide concern for human health, which was consist of particulate matter (PM) and gaseous pollutants. Fine particulate matter (PM_2.5_) is considered the most important pathogenic PM with an aerodynamic diameter ≤ 2.5 microns 1. Due to its small volume but large surface area, PM_2.5_ may carry various toxic substances into the end of the respiratory tract with airflow and damage the airway epithelial barrier 2.

Recent epidemiological data have shown that PM_2.5_ is closely correlated with the high respiratory morbidity, especially in asthma and chronic obstructive pulmonary disease 3-7. Studies in China, America and European countries have demonstrated that PM_2.5_ can lead to higher hospital visit rates, increased hospitalization rates, all-cause mortality of respiratory diseases and decreased life expectancy 8-10. In short, the respiratory harmfulness of PM_2.5_ has raised worldwide concern. However, the mechanisms of PM_2.5_ on respiratory diseases remain a challenge.

Some studies revealed that PM_2.5_ has the toxicological effects on bronchial epithelial cells through inflammatory responses and oxidative stress 11, 12. Disruption of the airway epithelium barrier can promote the transportation of inhaled PM_2.5_ to the subepithelial zone, causing airway inflammation and immune responses 13, 14. Recent research suggested that the occurrence and clinical manifestations of asthma are closely related to changes in the physical and barrier properties of epithelial cells 15. These studies indicated some relations between barrier dysfunction and PM_2.5_-mediated airway epithelial injury, however, the specific molecular mechanisms still remain unknown.

Airway epithelial cells play a crucial role in the initial defense of the airways. They are covered with a thin liquid layer (~10 microns) called airway surface liquid (ASL), which is regulated through epithelial channel proteins, including the sodium channel and chloride channel 16. Cystic fibrosis transmembrane regulator (CFTR) is the chloride channel protein that plays a dominant role in anion transport 16, 17. In the airways, defective anion transport can impair mucociliary clearance 18. Recent studies revealed that CFTR dysfunction induced the intracellular hyperchloride and mucus accumulation, led to persistent inflammation and airway obstruction, and finally resulted in airway hyperresponsiveness in asthma model 19-21. A population study from America found that carriers with CFTR gene increased the risk of asthma 22. However, a systematic review revealed that the CFTR mutation had no significant relation with asthma susceptibility 23. Consequently, the relations between CFTR and asthma are still unclear.

Adenosine triphosphate (ATP) is a crucial signaling molecule for water-salt transport in airways 24. Previous study showed that ATP can increase the amount of ASL in airways, and activate the P2Y receptors to participate in proliferation and anti-inflammation 25-27. Some purinergic receptors had been identified as the highly expressed receptors in lung tissues, and may be the potential treatment targets in asthma 28. Additionally, P2Y2 receptor (P2Y2R) is the most important P2Y receptors for anion transport in airway epithelial cells 29. A study proved that P2Y2R is associated with murine lung allergic inflammation and Th2 response pathways 30. These studies indicates that ATP/P2Y2R participate in anion transport in airways, and P2Y2R may be associated with allergic inflammation. However, the relations of ATP/P2Y2R-dependent pathway on water-salt transport and asthma after PM_2.5_ exposures still remain unclear.

This research aimed at exploring the specific effects and potential mechanisms of PM_2.5_ on transepithelial anion short-circuit current *in vitro* and *in vivo*, and revealing the relations between PM_2.5_ and airway inflammation. We hoped to provide some novel insights on the PM_2.5_-induced asthma.

## Materials and Methods

### PM_2.5_ sampling and LC-MS/MS analysis

PM_2.5_ was obtained from Guangzhou Institute of Geochemistry, Chinese Academy of Sciences from July to November 2019. The PM_2.5_ samples were collected in a Tissuquartz (PALL, California, USA) through a high-flow PM_2.5_ sampler (Thermo Fischer Scientific, Waltham, USA) with the flow rate of 1.05 m^3^/min, and immediately stored at -20°C for further experiments. First, we cut the quartz fibre filter membrane into small pieces (5 cm × 5 cm) and put them into ultrapure water. Then, PM_2.5_ was eluted from the membrane with ultrasonic oscillation for 30 min three times. Third, the eluent was filtered through a sterile gauze, and centrifuged for 1 h at 4°C, 12000 r/min. We preserved the bottom layer solution of the eluate containing PM_2.5_ at -80°C. Fourth, the solution was freeze-dried in a vacuum condition for 24 h, and the PM_2.5_ sample was sterilized with ultraviolet light for 6 h. Finally, a mixed solution of 200 μg/mL were prepared with serum-free Dulbeccos modified Eagles medium/F12 (DMEM/F12, Gibco, USA). We measured and analyzed the water-soluble components of PM_2.5_ through LC-MS/MS technology, including total organic carbon (TOC) (Elementar total organic carbon analyzer, Germany), water-soluble metallic ions, inorganic salt anions and cations (Inductively coupled plasma mass spectrometer, Germany).

### Cell culture and proliferation

Human airway epithelial cells (Calu-3) were purchased from Procell Life Technology Co., Ltd (Wuhan, CN). Calu-3 cells were cultured in submerged monolayer conditions, in order to simulate the air-liquid interface in transwell plates (3801, Corning, USA). Dulbecco's modified eagle medium (DMEM) (Gibco, USA) containing 10% fetal bovine serum (Thermo Fisher, Shanghai, CN) and 1% penicillin G/streptomycin (NCM biotech, Suzhou, CN) was used to culture cells for 21-day post-confluency prior to PM_2.5_ exposure at 37°C and 5% CO_2_ in an incubator. Then Calu-3 cells were exposed to PM_2.5_ or ATP (Sigma, USA) treatment for 24 h, and the control was added with an equal volume of PBS (Corning, USA). Finally, these samples were used to detect transepithelial I_sc_ or ASL.

Furthermore, Calu-3 cells were incubated in six-well plates to explore the potential molecular mechanism. This experiment was divided into six groups, including control group, ATP (10 μM) group, PM_2.5_ (100 μg/mL), Suramin (100 μM) (Sigma, USA) group, ATP+PM_2.5_ group and Suramin+PM_2.5_ group. Calu-3 cells were bathed with DMEM/F12 medium (containing 10% fetal bovine serum and 1% penicillin/streptomycin) for 24 h and 48 h. We measured the expression levels of P2Y2R and CFTR through quantitative real-time polymerase chain reaction (qRT-PCR) and western blot.

### Establishment of OVA-induced asthmatic mice

A total of 50 male Balb/c mice, aged 6-8 weeks, weighing 18-22 g, SPF grade were purchased from Hunan Slake Jingda company (Hunan province, CN). All animal experiments were conducted in the Animal Research Center of South China University of Technology (License No.: SYXK (Guangdong) 2017-0178). This study was approved by the Research Ethics Committee of Guangdong Provincial People's Hospital (approval No.: GDREC2019347A). 50 mice were randomly divided into 5 groups: control group, OVA group, OVA+ATP group (ATP, 50 mg/kg), OVA+PM_2.5_ group (PM_2.5,_ 100 μg/mL), OVA+Suramin group (Suramin, 50 mg/kg). In experimental groups, mice were intraperitoneally injected with OVA (0.5 mg/kg) (Macklin, CN) plus aluminum hydroxide (50 mg/kg) (Macklin, CN) for sensitization on day 1, 7 and 14, and then were inhaled with 1% OVA once per day for challenge from day 21 to 27. Besides, mice were given nasal instillation of PM_2.5_, ATP and Suramin solution from day 21 to 27 in OVA+PM_2.5_ group and OVA+ATP group, OVA+Suramin group, respectively. In control group, mice were intraperitoneally injection and inhaled with equal volume of saline. On day 28, mice were euthanized to acquire samples for subsequent experiments.

### Airway surface liquid layer

In order to investigate the effects of PM_2.5_ on airway epithelial barriers, we detected the volume of ASL after PM_2.5_ exposure. ATP was the activator of P2Y2R, therefore this experiment included control, ATP group (10 μM), PM_2.5_ group (100 μg/mL) and ATP+PM_2.5_ group. When cells grown up to confluency of 100% in transwell plates, 100 μL (ATP and PM_2.5_ were dissolved in medium) solution were added into each group. The control group was pre-treated with 100-μL PBS. The volume of ASL were measured on the chamber through a pipette with an accuracy of 0.01 μL after 24 h. This experiment was repeated three times.

### K-H solution preparation

K-H solution was prepared for electrophysiological experiment 31. K-H solution included solution A and solution B according to different components. Solution A is composed of sodium chloride (120 mM), sodium bicarbonate (25 mM), potassium dihydrogen phosphate (3.3 mM), dipotassium hydrogen phosphate (0.128 mM), magnesium chloride (1.2 mM), calcium chloride (1.2 mM), and mannitol solution (10 mM). However, solution B is similar to solution A, differently the mannitol is replaced with glucose solution (10 mM).

### TER and ATP-induced transepithelial anion I_sc_

To reveal the effects of PM_2.5_ exposure on TER and ATP-induced transepithelial anion I_sc_. Firstly, we measured the TER with RE1600 epithelium resistor (Kingtech, Beijing, CN). Secondly, we loaded the samples on the ussing chamber of electrophysiological detection equipment (Kingtech, Beijing, CN). Thirdly, we added 5-ML K-H solution into ussing chamber which was composed of side A and side B, and solution A and B corresponded to side A and B, respectively. Fourth, we prepared current and voltage electrodes which composed of a pipette-like tip and a small volume agarose gel. The agar gel (1%, dissolved in 3 M potassium chloride solution) was perfused into the electrode tip (length: 5-10 mm), and potassium chloride solution (3 M) was added into the other end of electrode. Besides, we needed to close the electrode head using cover with wires, and the electrodes were inserted into Ussing chamber to connect with electrophysiological detection equipment. Fifth, we checked the electrophysiological detection equipment at 37°C, 95% oxygen and 5% carbon dioxide. If the current was above 60 μA, it meant that the electrode was available, and adjusted the resistance difference of solution A and B to be zero by resistance compensation. Instantly, we connected computer program, set the parameter of voltage as zero, and started to collect data. When the baseline current was stable after several to 30 min, we needed recorded the baseline data of I_sc_. It is worth nothing that the balanced time or baseline I_sc_ might have been different in each group. After the baseline I_sc_ was stable, ATP solution (10 μM) was added into side A to induce I_sc_ peak in control group and PM_2.5_ group. We focused on analyzing the response speed of I_sc_ from baseline to peak value after ATP stimulation, including the maximum difference (∆I_sc_) and average rate of I_sc_. Average rate (∆I_sc_/∆T) refers to the ratio of the difference value from the baseline to maximum of I_sc_ (∆I_sc_) and the time (∆T) difference after the addition of the administration of ATP.

In the animal experiment, the specific protocol is as previously described 31, 32. In short, we first acquired the main trachea tissue with the area of 8 mm × 8 mm, and immediately put it into saline for keeping vitality. Then, we gently removed the blood vessels and connective tissue of the trachea tissue completely. Third, the trachea tissue was fixed on the tissue clip, and K-H solution was added into ussing chamber (solution A to the luminal side, solution B to the basal side). Finally, the following protocols were conducted as the cell experiment. Differently, the concentration of ATP solution was 100 μM.

### Real-time qRT-PCR

The P2Y2R/CFTR pathway and intracellular calcium play a key role in anion transport of airway epithelial cells 17, 29. Therefore, we assessed the levels of P2Y2R, CFTR and intracellular calcium after PM_2.5_ exposure. Total RNA was extracted from Calu-3 cells by TRIzol method (Sigma, USA). The cDNA was synthesized from total RNA using the reverse transcription kit (Takara, Dalian, CN). The expression levels of P2Y2R were measured by qRT-PCR according to manufacturer's instructions. GADPH was used as an internal control. P2Y2R, forward primer: CCTGAGAGGAGAAGCGCAG, reverse primer: GAACTCTGCGGGAAACAGGA. GADPH, forward primer: AGATCCCTCCAAAATCAAGTGG, reverse primer: GGCAGAGATGATGACCCTTTT. Repeat this experiment three times.

### Western blot

RIPA lysate (Solarbio, Beijing, CN) was used to extract total protein from Calu-3 cells and right lung tissues of mice. Total protein was quantified through the BCA Protein Assay Kit (Beyotime, Shanghai, CN). Total protein (40 μg/lane) was separated using 10% sodium dodecyl sulfate-polyacrylamide gel electrophoresis (SDS-PAGE) (Beyotime, Shanghai, CN). Then, the protein was transferred to a polyvinylidene difluoride (PVDF) membrane (Millipore, Billerica, MA, USA). The membranes were blocked in 5% skim milk/Tris-buffered saline Tween (TBS-T) (Beyotime, Shanghai, CN) for 1.5 h. Subsequently, they were probed with specific primary antibodies against P2Y2R (Abcam, ab272891, 1:1000) and CFTR (CST, 78335S, 1:1000). Secondary antibody anti-rabbit IgG (CST, 7074S, 1:2500) with horseradish peroxidase (HRP)-labeled was incubated for 2 h at room temperature. A ChemiScope series (Clinx, Shanghai, CN) was used to observe immunoreactive bands, and protein bands were subject to semi-quantitative analysis using densitometry. Repeat this experiment three times.

### Flow cytometry assay

We measured the cytoplasmic free calcium using flow cytometry assay as previously described 33. Firstly, Calu-3 cells were collected and digested with trypsin without EDTA. Secondly, the cells were washed with the calcium-free PBS twice. Thirdly, each sample was equally divided into two halves, one of which was labeled with 0.5 μL Fluo-3/AM fluorescent probe (Beyotime, Shanghai, CN), and another half was the control one. Fourth, all samples were incubated at 37°C for 30 min, and were washed with calcium-free PBS twice. Subsequently, cells were resuspended with 400 ul PBS buffer and incubated for 20 min. Lastly, the fluorescence of FITC channel was measured through flow cytometer (BD Accuri^®^ C6 Plus, USA). The excitation wavelength of Fluo-3/AM probe is 488 nm, and the emission wavelength is 525 nm. FlowJo software (Version 10.0, USA) was used to analyze the mean fluorescence intensity, and the experiment was repeated three times.

### Lung function test

Airway hyperresponsiveness is an important characteristic of asthma 34. To reveal the relationship of P2Y2R and airway hyperresponsiveness, we determined the airway resistance of mice after inhalation of methacholine. First, we placed mice into the whole-body chamber and connected to the computer program (BUXCO, DSI, MN, USA). The airway responsiveness was expressed with special airway resistance (sRaw, cmH_2_O/mL/s) value. The sRaw value was obtained with aerosols containing different doses of methacholine (0, 5, 10, 20 mg/mL), and was analyzed with FLexi Vent software.

### Measurement of IL-4, IL-5 and IL-13 in murine BALF

Th2-related airway inflammation is the common feature of asthma 35. We measured Th2-related inflammatory cytokines (IL-4, IL-5 and IL-13) in BALF of mice to clarify the relationship between P2Y2R and airway inflammation. First, we ligated the left main bronchus with surgical thread after anesthesia with the inhalation of isoflurane (RWD, Guangzhou, CN). Then, the right lung was washed with 0.5 mL saline twice, and the lavage liquid was collected. Lastly, the expression levels of inflammatory cytokines of BALF were detected using mouse ELISA kits (Absin, Shanghai, CN). The operative protocols are as the manufacturer's instructions.

### HE, PAS and Masson staining

Hematoxylin and eosin (HE) (Sigma, St.Louis, USA) staining was performed to evaluate the state of lung inflammatory, tissue edema and epithelial damage. Tissue slices (5 μm) were stained with Periodic Acid-Schiff (PAS) to assess the status of hyperplasia of goblet cells in the bronchus. Besides, Masson staining was selected to display collagen fibers. The Smith scoring system of lung injury was employed for semi-quantitative analysis. Two pathologists blindly assessed 10 lung tissues for injury severity on HE staining, including four sections: pulmonary edema; alveolar and interstitial inflammation; alveolar and interstitial hemorrhage; atelectasis and the formation of hyaline membranes. In addition, the tissue lesions were scored as following: 0 (no lesion); 1 (0-25%); 2 (25%-50%); 3 (50%-75%); 4 (>75%). Thus, the lung injury total score is the sum of the 4 parameters. PAS and Masson staining were expressed in terms of the percentage of positive cells.

### Immunohistochemistry

The left lung was fixed with 4% paraform aldehyde (Sigma, St. Louis, USA) for 24 h. Tissues were paraffin-embedded (Sigma, St. Louis, USA) and cut into 5-μm slices. For immunohistochemical staining, lung tissue sections were incubated with specific primary antibodies against P2Y2R (1:500, Abcam, UK) and CFTR (1:1000, Abclone, CN). Sections were washed using PBS, and incubated with mouse anti-rabbit IgG and a streptavidin peroxidase (SP) complex for 40 min at 37°C (ZSGB-BIO, Beijing, CN). The findings were visualized using a 0.05% diaminobenzidine (DAB) substrate. The expression levels of P2Y2R and CFTR were quantified by analyzing the staining intensity. The expression levels of P2Y2R and CFTR in immunohistochemical staining were expressed with the percentage of positive cells.

### Statistical analysis

In this study, GraphPad Prism (Version 5.01, GraphPad Software, USA) was conducted to draw figures and compute data. Continuous variables were expressed with mean ± standard deviation. T-test or nonparametric test was used to reveal the difference between two independent samples. For three or above samples, an ANOVA test was employed to show the difference among all groups. P-value *<* 0.05 was considered statistically significant.

## Results

### LC-MS/MS revealed main chemical constituents of PM_2.5_

The LC-MS/MS revealed the main chemical components of PM_2.5_, including constant elements, trace elements, water-soluble ion and total organic carbon (TOC). The top five constant elements were K, Ca, Na, Zn and Al, and the top five trace elements were Ba, Mn, Cu, Pb and As** (Table 1)**. In addition, the top five cations were NH_4_^+^, K^+^, Ca^2+^, Na^+^ and Mg^2+^, and the top five anions were SO_4_^2-^, NO_3_^-^, Cl^-^, NO_2_^-^, and Br^-^ **(Table 2)**. Finally, the concentration of total carbon was 337 μg/mg **(Table 2)**. In short, the constituents of PM_2.5_ that we used were similar to the American National Institute of Standard Technologies (NIST), in which the mass fraction of both organic and inorganic constituents is certificated by high-performance liquid chromatography 36.

### PM_2.5_ inhibited ASL and transepithelial anion I_sc_

**Figure 1:**
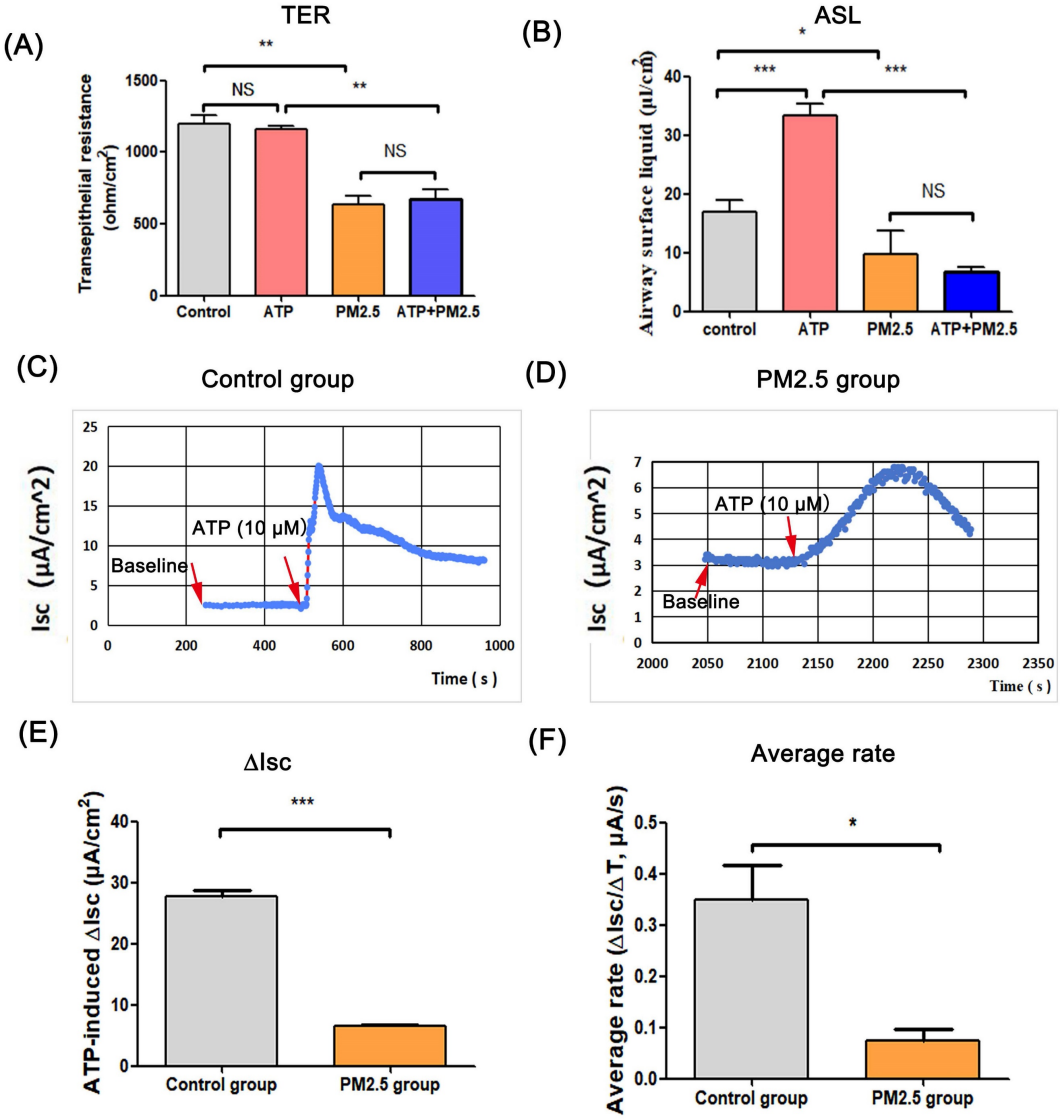
** The effects of PM_2.5_ on the transepithelial resistance, airway surface liquid and ATP-induced transepithelial short-circuit current in Calu-3 cells. (A)** Changes of the transepithelial resistance (TER) in all groups. **(B)** The amount of ATP-induced airway surface liquid in all groups. **(C)** The baseline level and ATP-induced I_sc_ peak in the control group. **(D)** The baseline level and ATP-induced I_sc_ peak in the PM_2.5_ group. **(E)** Changes of ATP-induced transepithelial short-circuit current (∆I_sc_) in the control group and PM_2.5_ group. **(F)** The average rate (∆I_sc_/∆T) of reaching I_sc_ peak from baseline. Red arrows: the time point of administration of ATP or baseline value. ASL, airway surface liquid. I_sc_, short-circuit current. PM_2.5_, fine particulate matter. ATP, adenosine triphosphate. Error bars correspond to 95% confidence intervals. One-way analysis of variance, NS: no significance, ^*^p < 0.05, ^**^p < 0.01, ^***^p < 0.001.

**Figure 2:**
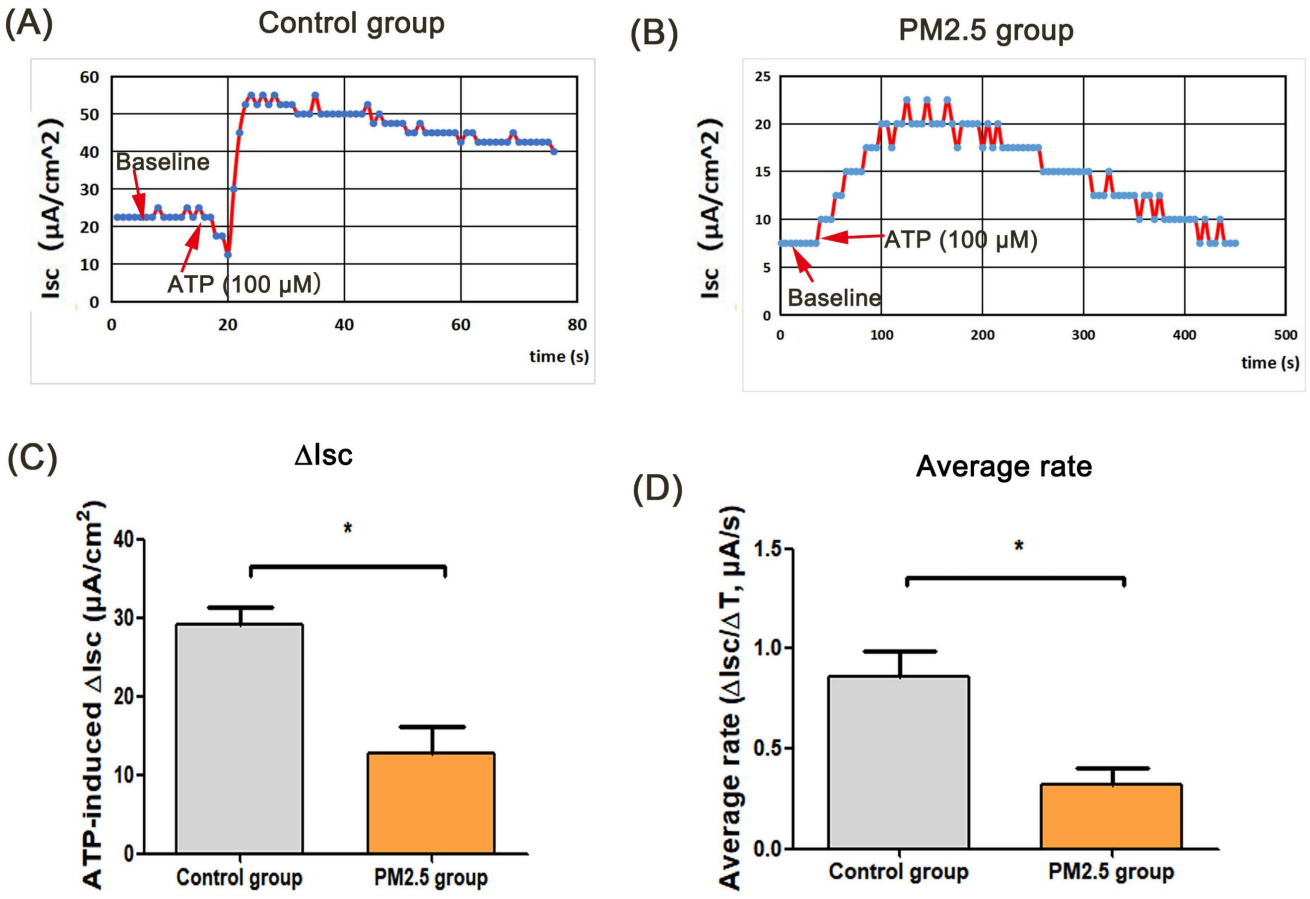
** The effects of PM_2.5_ on ATP-induced trans-tracheal tissue in OVA-induced asthmatic mice. (A)** The baseline level and ATP-induced I_sc_ peak in the control group. **(B)** The baseline level and ATP-induced I_sc_ peak in the PM_2.5_ group. **(C)** Changes of ATP-induced I_sc_ (∆I_sc_) on mouse tracheal tissue in the control group.** (D)** The average rate (∆I_sc_/∆T) of reaching peak from baseline. Red arrows: the time point of administration of ATP or baseline value. PM_2.5_, fine particulate matter. ATP, adenosine triphosphate. I_sc_, short-circuit current. Error bars correspond to 95% confidence intervals. Student's T test, ^*^p < 0.05.

ASL is the important airway barrier for defensing external pathogens, which is regulated by epithelium anion transport system 16. In this study, there was no statistical difference in TER between the ATP group and the control group, and the TER value in PM_2.5_ group was lower than that in the control group. Besides, the TER in PM_2.5_+ATP group was significantly lower than that in the ATP group **(Fig. 1A)**. In the transwell model, the volume of ASL in the ATP group was higher than that in the blank control group (p < 0.001). However, the amount of ASL in the ATP+PM_2.5_ group was evidently lower than that in the ATP group **(Fig. 1B)**. These results indicated that PM_2.5_ treatment reduced the ASL production in airway epithelial cells. Importantly, a stable baseline level of I_sc_ (no I_sc_ peak) was detected in the control group and PM_2.5_ group before ATP stimulation **(Fig.1C-D)**. Excitingly, there was a rapidly rising I_sc_ peak (called ATP-induced I_sc_ peak) in the control group and PM_2.5_ group after the administration of ATP **(Fig. 1C-D)**. However, an increase (∆I_sc_) in the ATP-induced I_sc_ peak in the PM_2.5_ group was significantly slower than that in the control group (p <0.05)** (Fig. 1D)**. Obviously, compared with the control group, the average rate (∆I_sc_/∆T) of I_sc_ changes evidently decreased in the PM_2.5_ group **(Fig. 1E)**. Similarly, these findings could be obtained from the trachea tissue of mice **(Fig. 2)**. In short, PM_2.5_ exposures impaired the production of ASL and transepithelial anion I_sc_.

### PM_2.5_ downregulated the levels of P2Y2R/CFTR and cytoplasmic free calcium

**Figure 3:**
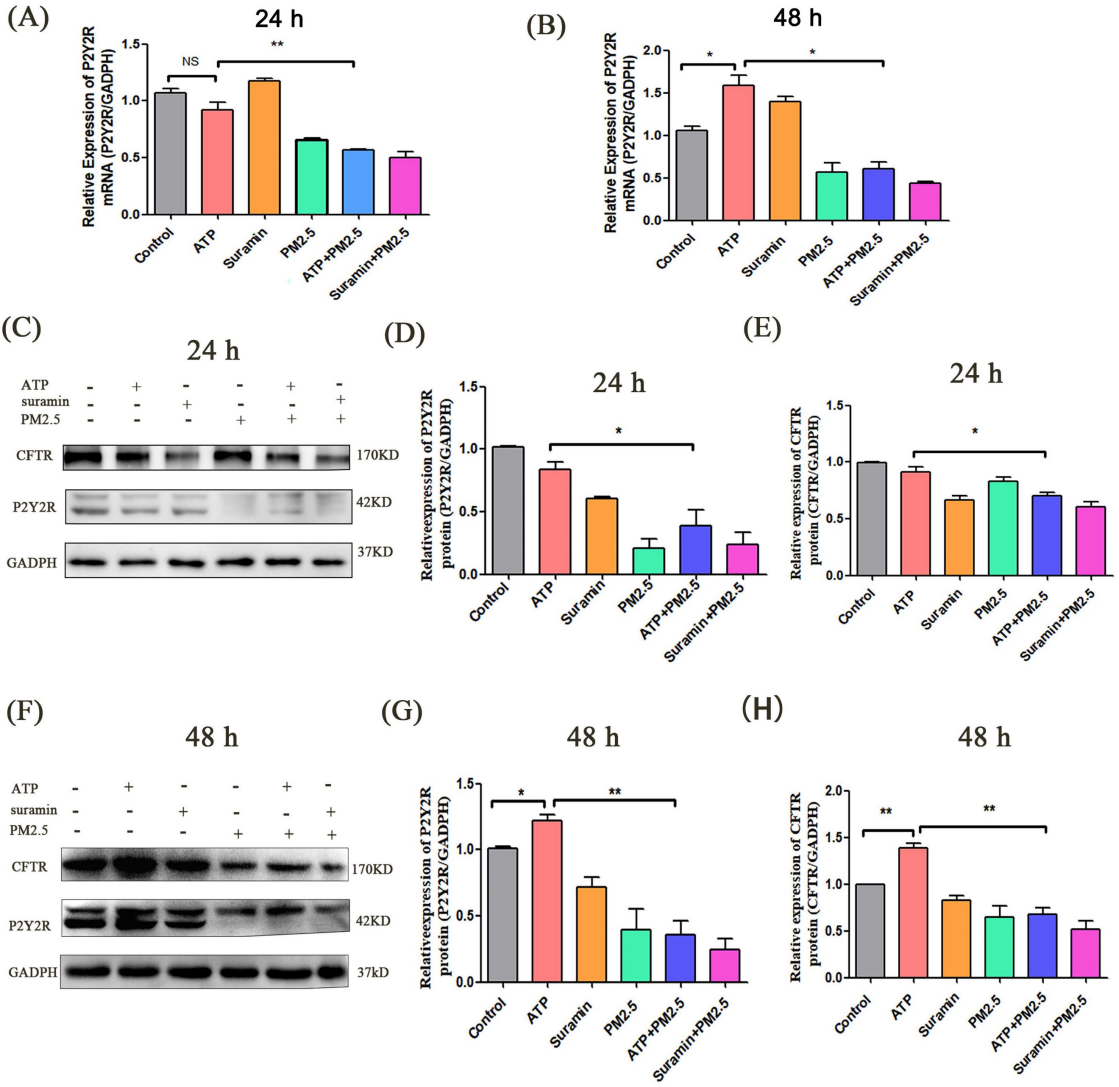
** The expression levels of P2Y2R/CFTR pathway after PM_2.5_ exposure in airway epithelial cells. (A)** Relative expression levels of *P2Y2R* mRNA in all groups after 24 h. **(B)** Relative expression levels of *P2Y2R* mRNA in all groups after 48 h. **(C)** Western blot analyses of proteins levels of P2Y2R and CFTR in Calu-3 cells after 24 h. **(D). (E)** Semi-quantification of densitometry of P2Y2R and CFTR. **(F)** Western blot analyses of proteins levels of P2Y2R and CFTR in Calu-3 cells after 48 h. **(G). (H)** Semi-quantification of densitometry of P2Y2R and CFTR. P2Y2R, P2Y2 receptor. CFTR, cystic fibrosis transmembrane regulator. PM_2.5_, fine particulate matter. ATP, adenosine triphosphate. Error bars correspond to 95% confidence intervals. One-way analysis of variance, NS: no significance, ^*^p < 0.05, ^**^p < 0.01.

The P2Y2R/CFTR pathway and intracellular free calcium play a key role in anion transport of airway epithelial cells 17, 29, 37, 38. In this experiment, the expression levels of *P2Y2R* mRNA had no difference between ATP group and control group at 24 h (p > 0.05) **(Fig. 3A)**. However, the expression levels of *P2Y2R* in the ATP group were evidently higher than that in the control group after 48 h. The expression levels of *P2Y2R* in the ATP+PM_2.5_ group were significantly lower than that in the ATP group. The expression levels of P2Y2R were lower in the PM_2.5_+Suramin group than that in the Suramin group** (Fig. 3B)**.

*In vitro*, western blot indicated that the expression levels of P2Y2R and CFTR significantly decreased after PM_2.5_ exposure. The expression levels of P2Y2R and CFTR in the Suramin group and PM_2.5_ group were significantly lower than these in the control group after 24 h and 48 h (p < 0.05) (**Fig. 3C-H**). Compared with the control group, the expression levels of P2Y2R and CFTR in the ATP group were elevated after 48 h (p < 0.05)** (Fig. 3F-H)**. In addition, the expression levels of P2Y2R and CFTR in ATP+PM_2.5_ group were significantly lower than that in the ATP group after 24 h and 48 h (p < 0.01)** (Fig. 3C-H)**. Interestingly, the protein levels of P2Y2R/CFTR in PM_2.5_+Suramin group were obviously lower than these in the Suramin group** (Fig. 3C-H)**.

**Figure 4:**
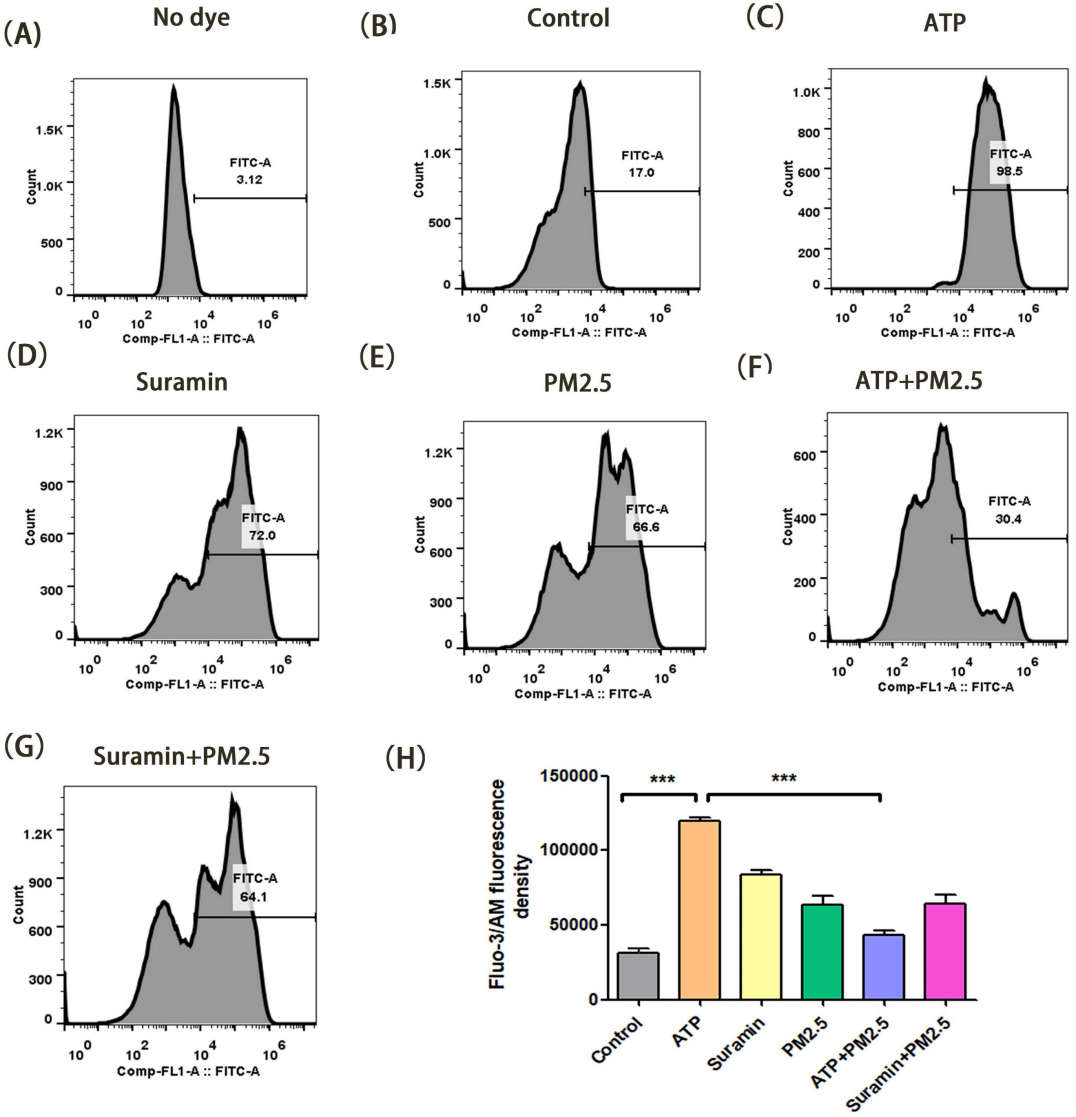
** Changes of cytoplasmic free-calcium in calu-3 cells after PM_2.5_ pre-treament for 24 h using a flow cytometry assay. (A)** The fluorescence density of cytoplasmic free-calcium in blank control group (No dye). **(B-G)** The fluorescence density of cytoplasmic free-calcium in all groups. **(H)** The data are expressed in terms of mean Fluo-3/Am fluorescence density. PM_2.5_, fine particulate matter. ATP, adenosine triphosphate. Error bars correspond to 95% confidence intervals. One-way analysis of variance, ^***^p < 0.001.

**Figure 5:**
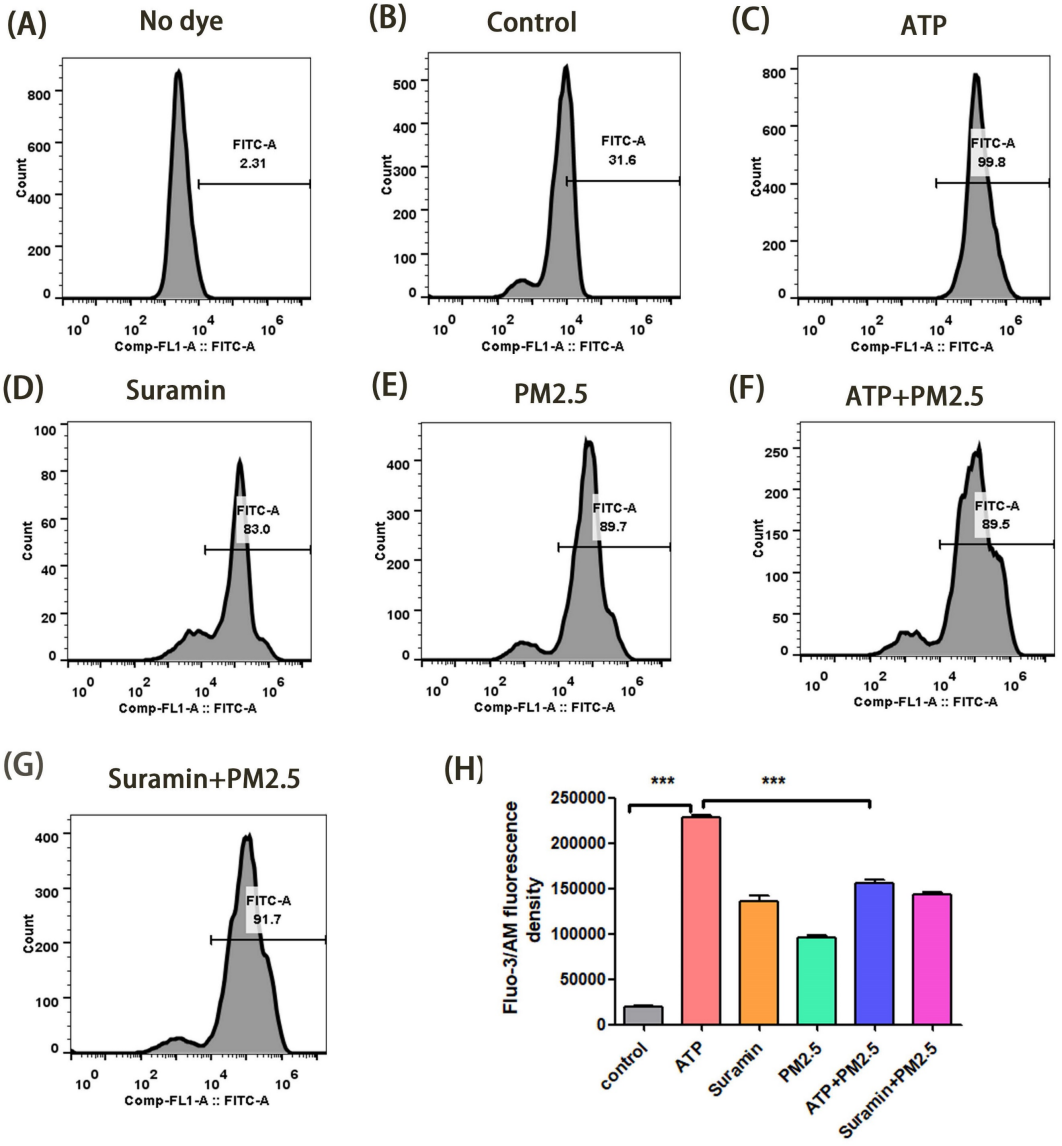
** Changes of cytoplasmic free-calcium in calu-3 cells after PM_2.5_ pre-treatment for 48 h using a flow cytometry assay. (A)** The fluorescence density of cytoplasmic free-calcium in blank control group (No dye). **(B-G)** The fluorescence density of cytoplasmic free-calcium in all groups. **(H)** The data are expressed in terms of mean Fluo-3/Am fluorescence density. PM_2.5_, fine particulate matter. ATP, adenosine triphosphate. Error bars correspond to 95% confidence intervals. One-way analysis of variance, ^***^p < 0.001.

In transwell model, we verified the changes of cytoplasmic free-calcium through a flow cytometry assay. The mean fluorescent intensity in the ATP group was significantly higher than that of the control group after 24 h and 48 h (p <0.01), but the fluorescent intensity in the ATP+PM_2.5_ group were significantly lower than that of the ATP group (p < 0.01) **(Fig. 4-5)**. Our study demonstrated that PM_2.5_ inhibited the release of ATP-induced cytoplasmic free calcium in the airway epithelial cells.

**Figure 6:**
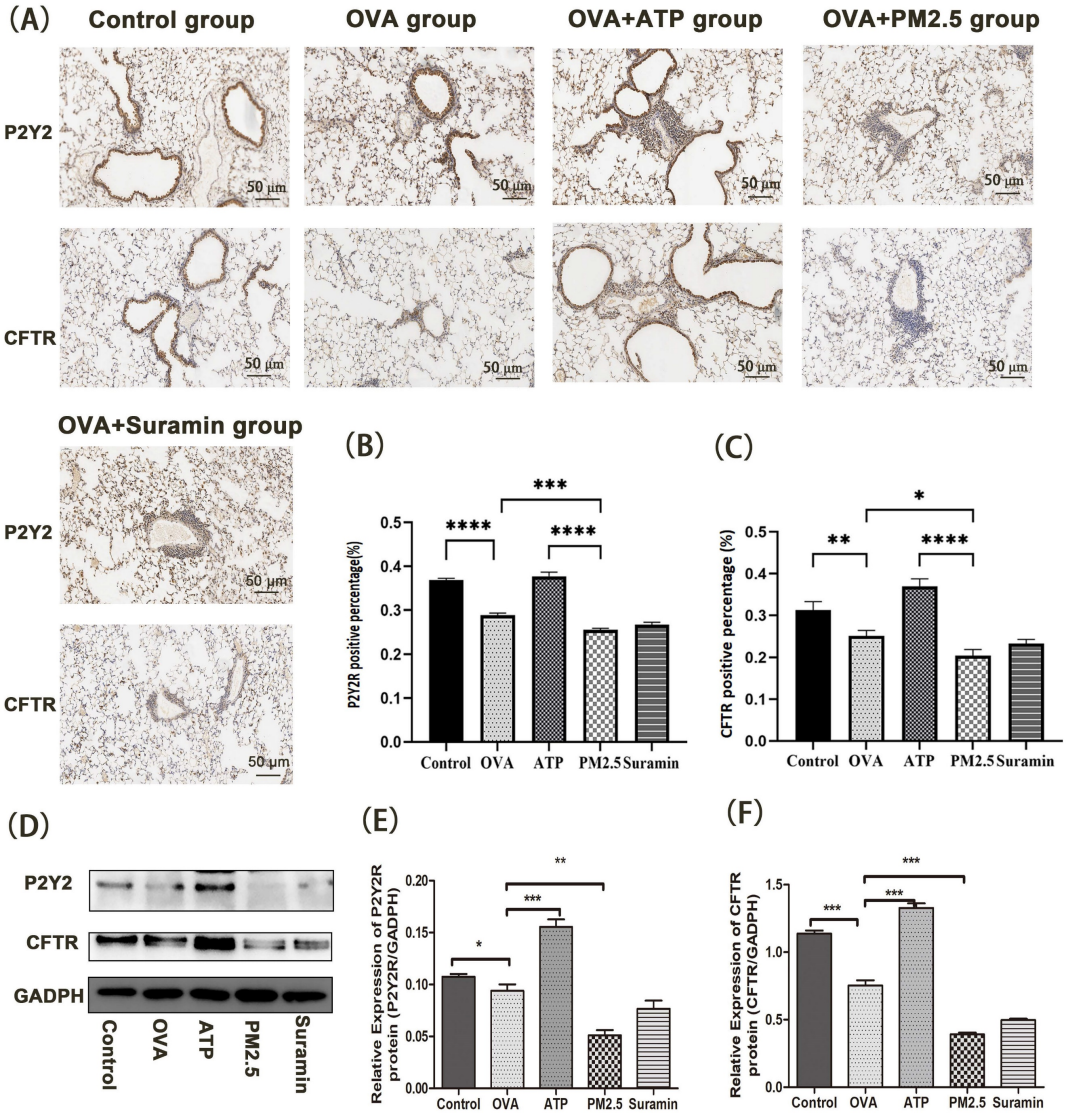
** The effects of PM_2.5_ on P2Y2R/CFTR pathway in lung tissues of OVA-induced mice using immunohistochemical staining and western blot (Scale bar: 50 μm). (A)** The expression levels of P2Y2R and CFTR protein using immunohistochemical staining. **(B)** The data were expressed in terms of P2Y2R positive area percentage of all groups.** (C)** The data were expressed in terms of CFTR positive area percentage of all groups. **(D)** The expression levels of P2Y2R and CFTR protein in each group using western blot. **(E)** The Quantification of densitometry of P2Y2R protein bands. **(F)** Quantification of densitometry of CFTR protein bands. GADPH was a loading control. P2Y2R, P2Y2 receptor. CFTR, cystic fibrosis transmembrane regulator. PM_2.5_, fine particulate matter. ATP, adenosine triphosphate. OVA, ovalbumin. Error bars correspond to 95% confidence intervals. One-way analysis of variance, ^*^p < 0.05, ^**^p < 0.01,^ ***^p < 0.001, ^****^p < 0.0001.

In animal experiments, we further examined the expression levels of P2Y2R/CFTR pathway using immunohistochemistry and western blot **(Fig. 6).** Compared with control group, the expression levels of P2Y2R and CFTR in the OVA group dramatically decreased. Furthermore, the expression levels of P2Y2R and CFTR in the PM_2.5_ group were significantly lower than these in the OVA group. However, compared with the OVA group, the expression levels of P2Y2R and CFTR in the ATP group significantly increased.

In summary, these findings showed that PM_2.5_ exposure downregulated the levels of P2Y2R/CFTR pathway and cytoplasmic free calcium.

### PM_2.5_ increased the airway responsiveness of mice

**Figure 7:**
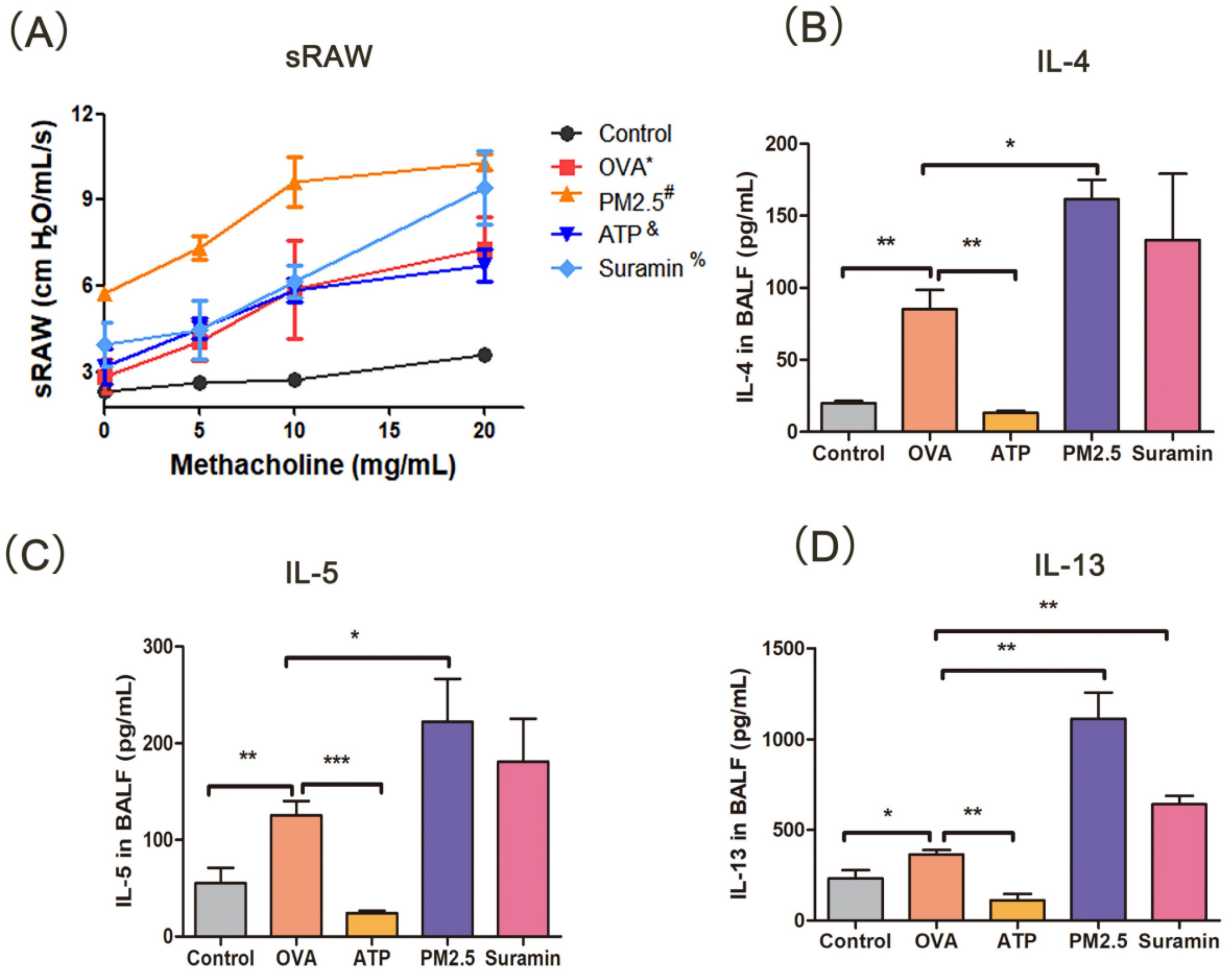
** Changes of specific resistance airways and Th2-related cytokines (IL-4, IL-5 and IL-13) with pre-treatment in mouse model. (A)** Changes of specific resistance airways (sRaw) in all groups after aerosol inhalation of increasing concentration of methacholine. **(B-D)** The expression levels of IL-4, IL5 and IL-13 of BALF in murine model. sRAW, special airway resistance. PM_2.5_, fine particulate matter. ATP, adenosine triphosphate. OVA, ovalbumin. BALF, bronchoalveolar lavage fluid. IL, interleukin. Error bars correspond to 95% confidence intervals. OVA^*^, p <0.05 compared with control group. #, & and %, p <0.05 compared with OVA group. One-way analysis of variance, ^*^p < 0.05, ^**^p < 0.01, ^***^p < 0.001.

PM_2.5_ is closely correlated with airway hyperresponsiveness, which is an important feature of asthma 34. In this experiment, with the increasing concentration of methacholine, the OVA-induced mice occurred restlessness and ragged breathing, but the mice in the control group had no above-mentioned manifestation. The sRaw of OVA-induced asthma mice increased significantly than that in the control group, suggesting that the asthmatic model was successfully constructed. The sRaw value in PM_2.5_ group was significantly elevated than that in the OVA group. The sRaw value of methacholine inhalation at 20 mg/mL in the Suramin+OVA group was higher than that in the OVA group alone (P <0.05). The sRaw value in the ATP group had no difference with the OVA group **(Fig. 7A)**. These results demonstrated that P2Y2R may be an important target for airway responsiveness in asthma model.

### PM_2.5_ aggravated lung inflammation, collagen deposition and hyperplasia of goblet cells

**Figure 8:**
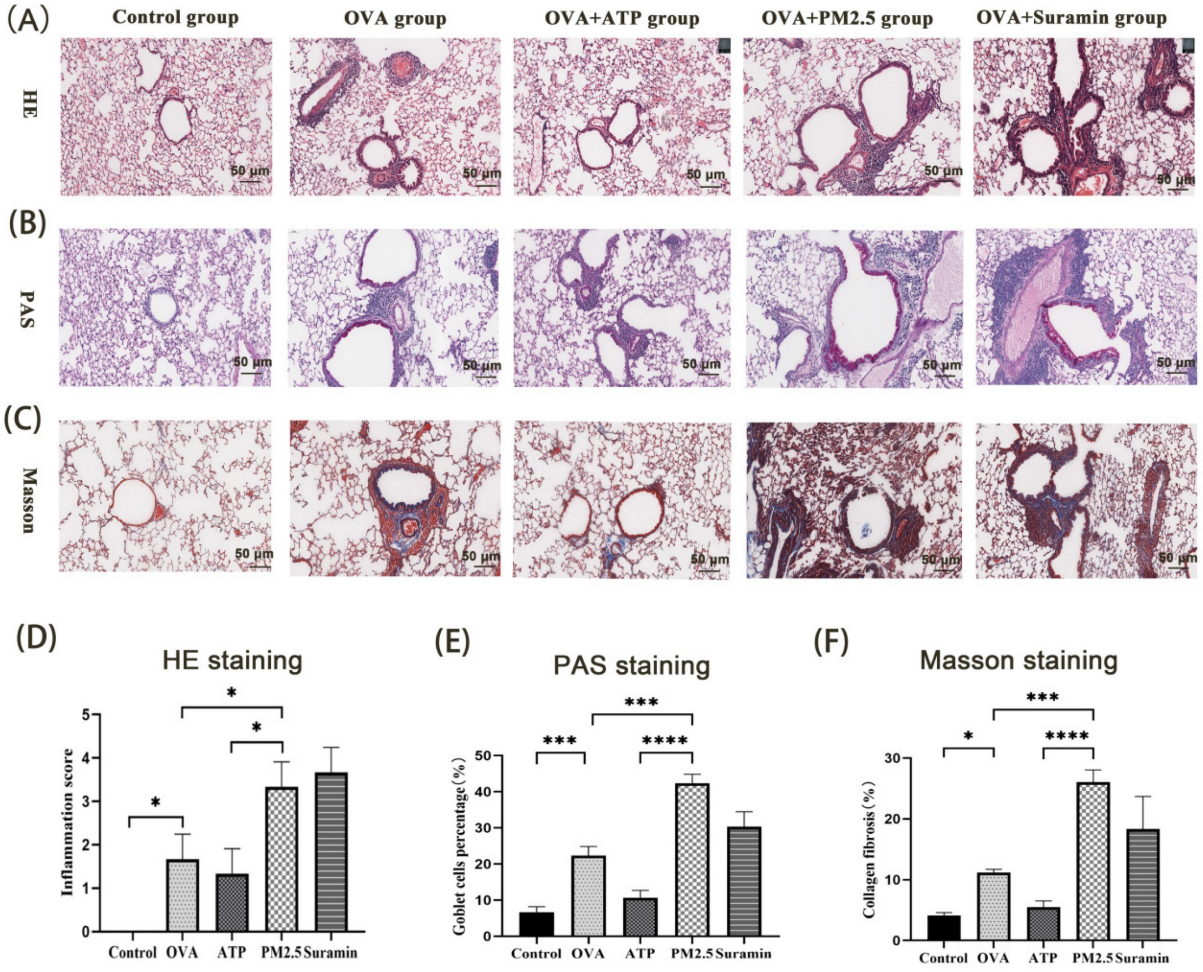
** The effects of PM_2.5_ on lung inflammation, hyperplasia of goblet cells and collagen deposition in OVA-induced asthma using HE, PAS and Masson staining. (A)** Representative images of lung inflammation through HE staining. **(B)** Representative images of hyperplasia of goblet cells in bronchus through PAS staining. **(C)** Representative images of collagen deposition in lung tissues through Masson staining. **(D)** Semi-quantitative analysis to HE staining by Smith score. **(E)** Semi-quantitative analysis to PAS staining in terms of goblet cell positive percentage (%). **(F)** Semi-quantitative analysis to Masson staining in terms of collagen percentage (%). PM_2.5_, fine particulate matter. ATP, adenosine triphosphate. OVA, ovalbumin. Error bars correspond to 95% confidence intervals. Non-parametric test, ^*^p < 0.05, ^***^p <0.001, ^****^p < 0.0001.

Th2-related airway inflammation is the common feature of asthma 35. In this study, the expression levels of IL-4, IL-5, and IL-13 in the OVA group were higher than these in the control group. Compared with OVA group, the expression levels of IL-4, IL-5 and IL-13 in PM_2.5_ group significantly increased. The levels of IL-4, IL-5 and IL-13 in the ATP group were lower than these in the OVA group **(Fig. 7B-D)**, but the expression levels of IL-13 in the Suramin group were higher than that in the OVA group **(Fig. 7D)**. For HE staining, the mice of the control group had normal alveolar morphology. In OVA group, the integrity of alveolar structure was destroyed, with thick alveolar septum, pulmonary artery wall and an increase in inflammatory cells **(Fig. 8A)**. Compared with the OVA group, the infiltration of lung inflammatory cells in ATP group was obviously reduced, and mice in PM_2.5_ group and Suramin group had higher Smith score **(Fig. 8D)**.

The proportion of goblet cells in trachea and bronchus is usually used to assess the mucus hypersecretion in asthmatic mice. Tissue Cells stained by PAS were regarded as goblet cells, which were mianly distributed near trachea and bronchus** (Fig. 8B)**. Compared with the blank group, the proportion of goblet cells was significantly higher in the OVA mice. The proportion of goblet cells in the ATP-treated OVA mice was evidently lower than that in the OVA group. However, the proportion of goblet cells in PM_2.5_ group and Suramin group was obviously higher than that in OVA group **(Fig. 8E)**. For Masson staining, compared with the control group, the OVA group showed different degrees of collagen deposition in the lung interstitium **(Fig. 8C)**. The area of collagen fiber deposition in the ATP group was lower than that of OVA group, but PM_2.5_ group and Suramin group had opposite tendency **(Fig. 8F)**. This evidence suggested that P2Y2R was closely related to lung inflammation in asthma model.

## Discussion

In this study, we clarified a potential mechanism through which PM_2.5_ exposure impairs airway epithelial anion-water transport and aggravates airway inflammation. Furthermore, PM_2.5_ exposure reduced the ATP-induced epithelial anion short-circuit current by downregulating P2Y2R/CFTR pathway *in vitro* and *in vivo*, and this process may participate in aggravating airway hyperresponsiveness and airway inflammation. These findings are likely linked to the pathogenesis of asthma after air pollution exposure.

Firstly, we investigated the effects on airway epithelial barriers after PM_2.5_ exposure, including ASL production and transepithelial I_sc_. We found that PM_2.5_ exposure affected the process of ATP-induced anionic short-circuit current and reduced the volume of ASL *in vitro*. *In vivo*, PM_2.5_ exposure similarly inhibited the ATP-induced transepithelial I_sc_ of tracheal tissue. We systematically reported the effects of PM_2.5_ on airway epithelial water-salt transport. Previous studies revealed that airway epithelial physiological functions were related to the development of asthma 39-42. In airways, the ASL plays a key role in the epithelial barriers, and water-salt transport can modulate the constituents of ASL. ATP is a crucial signaling molecule released by epithelial cells after external attack, and can activate P2Y2R to modulate the chloride ion secretion and water-salt transport in respiratory system, while the P2Y2R antagonist (Suramin) can inhibit this process 4, 37, 43. Tarran *et al.* 44. found that a lack of ATP inhibits the purinergic-dependent chloride ion outflow and reduces the height of ASL, leading to mucus obstruction. In this experiment, there was no peak value of I_sc_ in the blank control group, however the ATP-induced I_sc_ peak and average rate in the PM_2.5_ group was significantly lower compared with the ATP group. These indicators reflect how quickly the airway epithelial ion channels open 45. In our research, we inferred that the inhibitory effect of PM_2.5_ on ASL may be related to the partial inhibition of ATP-induced anionic current by the components of PM_2.5_. The anion current is mainly controlled by the ion channel CFTR of airway epithelial cells. In airways, the epithelial cells can sense local stress and release ATP immediately to induce anion outflow to increase the thickness of the liquid layer on the airway surface. Previous studies have shown that the ATP-induced anion current is mainly realized through the P2Y2R of the airway epithelium 46, 47, which was confirmed by further experiments. Besides, we have shown that inhibitors utilizing ENaC failed to reduce the anionic current peak, however, the CFTR function was significantly inhibited after replacing chloride ion into the glucuronide ion 31. In a word, these results indicated that PM_2.5_ exposure impaired the transepithelial I_sc_ of airway epithelial cells.

Next, we explored the relations between P2Y2R and airway hyperresponsiveness and lung inflammation after PM_2.5_ exposures *in vivo*. The results revealed that PM_2.5_ and Suramin exposures could increase the airway hyperresponsiveness, lung inflammatory, collagen fiber deposition and hyperplasia of goblet cells in OVA-induced asthma mice, whereas ATP administration reduced airway inflammatory in asthma mice. Previous studies showed that the inflammatory response is one of the main mechanisms of airway injury induced by PM_2.5_ 11, 12. Some studies clarified that intraperitoneal injection or nasal infusion of PM_2.5_ could aggravate airway eosinophil inflammatory infiltration, leading to increasing airway hyperresponsiveness and Th2-related cytokines in BALF 48, 49. P2Y2R is the most important P2Y receptor for anion transport in airway epithelial cells, and is associated with murine lung allergic inflammation and Th2 response pathways 29, 30. Importantly, Th2-ralated inflammation is the common and classic feature in asthma 35. Therefore, we evaluated the expression levels of Th2-related cytokines and airway responsiveness. In this study, the expression levels of IL-4, IL-5 and IL-13 in BALF of PM_2.5_+OVA group are evidently higher than these in the OVA group, which is consistent with previous study 49. Compared with OVA group, the expression levels of IL-4, IL-5 and IL-13 in the ATP group evidently decreased, but the IL-13 in the Suramin group was in lower level. The results in cytokines of BALF revealed that P2Y2R may be associated with Th2-related airway inflammation. In addition, the sRaw of OVA-induced asthma mice increased significantly than that in the control group, suggesting that the asthmatic model was successfully constructed. The sRaw value in PM_2.5_ group was significantly elevated than that in the OVA group. The sRaw value of methacholine inhalation at 20 mg/mL in the Suramin+OVA group was higher than that in the OVA group alone (p <0.05). This evidence demonstrated that P2Y2R may be an important target for airway responsiveness in asthma model.

Furthermore, PM_2.5_ exposure aggravated inflammatory infiltration and hyperplasia of goblet cells, as well as changes in lung ultrastructure in an asthmatic mouse model 50. Therefore, we also assessed the lung inflammation, hyperplasia of goblet cells, and collagen deposition in the lung tissues of the experimental mice by using HE, PAS, and Masson staining. The ATP administration depressed the lung inflammation, goblet cell hyperplasia and collagen deposition, but PM_2.5_ or Suramin exposures increased the lung airway inflammation. In short, PM_2.5_ exposure inhibited the ATP/P2Y2R signaling pathway of airway epithelial cells, leading to an increase in airway responsiveness and Th2-related inflammatory factors. This evidence suggested that PM_2.5_ may mediate acute airway inflammation by impairing the ATP-P2Y2R signaling pathway.

Finally, we explored the potential molecular mechanisms through which PM_2.5_ exposure reduced the transepithelial I_sc_ and exacerbated airway inflammation in OVA-induced asthma mice. Previous studies indicated that ATP can increase the levels of cytoplasmic free calcium to induce chloride ion outflow 37, 38, 51. Besides, ATP could bind to P2Y receptors to activate intracellular phospholipase C pathway, and evokes calcium ion signal 52. Therefore, intracellular free-calcium induced by exogenous ATP may be an important signaling molecule for chloride ion secretion. In this experiment, compared with the control group, ATP pre-treatment could induce significant elevation in cytoplasmic free-calcium, while Suramin or PM_2.5_ exposures significantly reduced the ATP-induced cytoplasmic free-calcium levels. These results suggested that the activation of calcium-dependent chloride channels may participate in modulating the transepithelial I_sc_ of airway epithelial cells.

CFTR is an anion transporter in epithelial cells, which allows chloride outflow 16, 17. Previous studies have found that ATP plays an important regulatory role in the activation of P2Y2R/CFTR pathway 53, 54. Nguyen 55 *et al.* reported tobacco smoke extract can reduce the expression and function of CFTR, however the urban PM (125 μg/mL) does not impact CFTR expression and dysfunction. Therefore, we examined the expression of P2Y2R/CFTR pathway in airway epithelium after PM_2.5_ exposure. In the air-liquid interface (transwell) model, we took 100 μg/mL of PM_2.5_ as the experimental condition according to preliminary experimental results 56. The results indicated that PM_2.5_ down-regulated the expression levels in mRNA and protein of P2Y2R, indicating that PM_2.5_ directly regulated the transcription process of P2Y2R and decreased the protein synthesis level. Previous studies have shown that Suramin is a specific antagonist of P2Y2R and can reduce the airway epithelial anion current 38. In our study, the degradation of P2Y2R was accelerated after the administration of Suramin, and the expression levels of P2Y2R was lower in the PM_2.5_+Suramin group than that in the Suramin group. Especially, we set co-treatment of Suramin and PM_2.5_ group to explore whether the two substances have a superimposed effect on P2Y2R. In our experiment, it was observed that the combination of the two substances has a more significant inhibition on expression levels of P2Y2R. Combining the above results, we inferred that P2Y2R may be the target of PM_2.5_ of airway epithelial anionic current. Furthermore, we also measured the expression levels of P2Y2R/CFTR pathway using western blot. Western blot showed that the expression levels of P2Y2R and CFTR were obviously elevated that in the control group, however, the Suramin or PM_2.5_ treatment downregulated the expression levels of P2Y2R and CFTR.

In the OVA-induced asthma model, we also found that Suramin or PM_2.5_ exposures increased the expression levels of P2Y2R and CFTR, and ATP treatment reduced the P2Y2R and CFTR expressions. Interestingly, this study reveals some positive results about P2Y2R/CFTR expressions and functions, and is different from previous study 55. It was worth nothing that the PM_2.5_ was collected unstandardised, and may lead to different experimental results. This variation might be due to the complex constituents of PM_2.5_, and we still need further experiments. Fu *et al.* explored that wood smoke PM_2.5_ could induce pyroptosis and inflammation of human bronchial epithelial cells through classic NLRP3-dependent and ATP/P2Y-dependent pathways 57. Regrettably, the relations between ATP/P2Y2-dependent pyroptosis and airway inflammation have been not reported in this study.

In this study, we explored the specific effects and potential mechanisms on impaired epithelial barrier functions from both *in vivo* and *in vitro* after PM_2.5_ exposure. Overall, the results of this study indicated that PM_2.5_ exposure inhibited the transepithelial anion I_sc_ by downregulating P2Y2R/CFTR pathway and P2Y2/calcium-dependent chloride channel, and this process may participate in aggravating the airway hyperresponsiveness, airway inflammatory, collagen deposition and hyperplasia of goblet cells. In contrast, ATP treatment appeared to mitigate these effects by upregulating P2Y2R and CFTR expression levels. This study provides valuable insights for PM_2.5_-mediated respiratory diseases, and first reported the relations between water-salt transport and airway inflammation after PM_2.5_ exposure. However, this study did not investigate the effects of specific constituents on the airway epithelial cells, which may be a limitation of this study.

## Conclusion

In summary, this study demonstrated that PM_2.5_ exposure impaired airway epithelial barriers through inhibiting ATP-induced anion I_sc_ by downregulating P2Y2R/CFTR pathway, and this process may participate in worsening airway hyperresponsiveness and airway inflammation. This study revealed that the P2Y2R may be a potential therapeutic target for asthma in clinic.

## Figures and Tables

**Table 1:** The constant element and trace element components of PM_2.5_

Constant Elements	Concentration (μg/mg)	Trace elements	Concentration (ng/mg)	Trace elements	Concentration (ng/mg)
K	54	Ba	1043	Cr	137
Ca	23	Mn	907	V	104
Na	17	Cu	866	Sr	90
Zn	6.0	Pb	732	Mo, Cd	67
Al	4.0	As	721	Co	61
Fe	3.7	Rb	200	Cs	39

Note: PM_2.5_, fine particulate matter.

**Table 2:** The water-soluble ion and total organic carbon components of PM_2.5_

Water-soluble anions	Concentration (μg/mg)	Water-soluble cations	Concentration (μg/mg)	Total organic carbon	Concentration (μg/mg)
SO_4_^2-^	739	NH_4_^+^	241	TOC	337
NO_3_^-^	124	K^+^	51		
Cl^-^	5.4	Ca^2+^	30		
NO_2_^-^	3.5	Na^+^	7.7		
Br^-^	2.3	Mg^2+^	2.1		

Note: TOC, total organic carbon. PM_2.5_, fine particulate matter.

## Abbreviations

PM_2.5_: Fine particulate matter
CFTR: Cystic fibrosis transmembrane regulator
P2Y2R: P2Y2 receptor
I_sc_: Short-circuit current
OVA: Ovalbumin
ATP: Adenosine triphosphate
ASL: Airway surface liquid
LC-MS/MS: Liquid chromatography-mass spectrometry/mass spectrometry
qRT-PCR: Quantitative real-time polymerase chain reaction
TER: Transepithelial resistance
SDS-PAGE: Sodium dodecyl sulfate-polyacrylamide gel electrophoresis
PVDF: Polyvinylidene difluoride
TBS-T: Milk/Tris-buffered saline Tween
HRP: Horseradish peroxidase
sRaw: Special airway resistance
HE: Hematoxylin and eosin
PAS: Periodic Acid-Schiff
SP: Streptavidin peroxidase
DAB: Diaminobenzidine
TOC: Total organic carbon
NIST: National Institute of Standard Technologies
BALF: Bronchoalveolar lavage fluid
IL: Interleukin
CaCC: calcium-dependent chloride channel
PIP2: phosphatidylinositol-4,5-bisphosphate
PLC: phospholipase C
IP3: inositol 1,4,5-trisphosphate
DAG: diacylglycerol
PKC: protein kinase C
